# Facile Synthesis of TiO_2_/MoS_2_ Composites with Co-Exposed High-Energy Facets for Enhanced Photocatalytic Performance

**DOI:** 10.3390/mi13111812

**Published:** 2022-10-24

**Authors:** Xianjun Niu, Yien Du, Jian Liu, Jinxiao Li, Jiayi Sun, Yuwei Guo

**Affiliations:** 1 Department of Chemistry and Chemical Engineering, Jinzhong University, Jinzhong 030619, China; 2 College of Chemistry, Baotou Teachers’ College, Baotou 014030, China

**Keywords:** crystal facets, molybdenum sulfide, titanium dioxide, TiO_2_/MoS_2_ composites, photocatalytic activity

## Abstract

In this work, with the the H_2_TiO_3_ colloidal suspension and MoS_2_ as the precursors, TiO_2_/MoS_2_ composites composed of anatase TiO_2_ nanocrystals with co-exposed {101} and [111]-facets (nanorod and nanocuboid), {101} and {010} facets (nanospindle), and MoS_2_ microspheres constructed by layer-by-layer self-assembly of nanosheets were hydrothermally synthesized under different pH conditions. The characterization has been performed by combining X-ray powder diffraction (XRD), field emission scanning electron microscopy (FESEM), transmission electron microscopy (TEM), high resolution TEM (HRTEM), X-ray photoelectron spectroscopy (XPS), photoluminescence (PL) spectra, and UV-visible absorption spectrum analyses. The photocatalytic degradation of rhodamine B (RhB) in an aqueous suspension was employed to evaluate the photocatalytic activity of the as-prepared pH*x*-TiO_2_/MoS_2_ composites. The photocatalytic degradation efficiency of pH3.5-TiO_2_/MoS_2_ composite was the highest (99.70%), which was 11.24, 2.98, 1.48, 1.21, 1.09, 1.03, 1.10, and 1.14 times that of Blank, MoS_2_, CM-TiO_2_, pH1.5-TiO_2_/MoS_2_, pH5.5-TiO_2_/MoS_2_, pH7.5-TiO_2_/MoS_2_, pH9.5-TiO_2_/MoS_2_, pH11.5-TiO_2_/MoS_2_, respectively. The pH3.5-TiO_2_/MoS_2_ composite exhibited the highest photocatalytic degradation rate, which may be attributed to the synergistic effects of its large specific surface area, suitable heterojunction structure, and favorable photogenerated charge-separation efficiency. This work is expect to provide primary insights into the photocatalytic effect of TiO_2_/MoS_2_ composite with co-exposed high-energy facets, and make a contribution to designing more efficient and stable photocatalysts.

## 1. Introduction

Over the last few decades, semiconductor photocatalysis technology has received more and more attention due to its potential applications in solving energy crises and reducing environmental pollution [1]. Up to now, many kinds of semiconductor photocatalysts have been developed, such as TiO_2_, ZnO, Cu_2_O, CdS, SrTiO_3_, C_3_N_4_, etc. [2,3]. Among them, TiO_2_ has been widely used in dye-sensitized solar cells, organic dye degradation, rechargeable lithium/sodium ion batteries, sensors, sunscreens, environmental remediation and other fields because of its excellent photocatalytic activity, chemical stability, inexpensiveness, abundance, innocuousness and non-toxicity [4,5]. However, the wide band gap, low charge separation rate, and rapid charge recombination rate of TiO_2_ limit its further practical application in the field of photocatalysis [6]. To overcome these shortcomings, various strategies have been developed to broaden the spectral response range of TiO_2_ and improve its photocatalytic efficiency, such as formation of semiconductor heterostructure, metal and nonmetal doping, noble metal deposition, morphology and crystal facets control, etc. [6,7,8]. Among these strategies, the construction of TiO_2_-based heterostructure semiconductors with high-energy facets by combining TiO_2_ with other semiconductors has attracted much attention because TiO_2_-based heterojunction with exposed high-energy facets can accelerate the transfer and separation of photo-induced charge carriers and enhance the photocatalytic performance [9].

Molybdenum disulfide (MoS_2_), a typical layered transitional metal dichalcogenide, has attracted a great deal of attention due not only to its tunable band gap energy (1.3–1.9 eV), thin thickness, and large surface area, but also to its abundant availability, low cost, high activity and special chemical stability [10,11,12]. Therefore, the TiO_2_/MoS_2_ heterojunction system formed by combining TiO_2_ and MoS_2_ with layered structure has high carrier separation and migration efficiencies, numerous reactive active sites, and a wide light-harvesting range, which can significantly enhance the photocatalytic activity [12]. In view of this, TiO_2_/MoS_2_ composites have attracted increasing interest in various catalytic applications related to environment and energy. For example, Shen et al. prepared MoS_2_ nanosheet/TiO_2_ nanowire hybrid nanostructures which exhibited enhanced hydrogen generation rate in visible light photocatalytic hydrogen evolution reaction [10]. Bai et al. prepared a TiO_2_/MoS_2_ hybrid structure which showed excellent performance for the photocatalytic hydrogen production and photocatalytic degradation of RhB [13]. Wang et al. prepared a MoS_2_/P25 composite photocatalyst which exhibited improved photocatalytic degradation activity for the degradation of rhodamine B (RhB) and methylene blue (MB) under simulated sunlight [14]. Zhang et al. prepared a 3D MoS_2_/TiO_2_ heterostructure by a simple hydrothermal method, which exhibited a higher photocatalytic performance for the degradation of RhB and methyl orange under UV light irradiation [15]. However, there are few reports on the synthesis of TiO_2_/MoS_2_ composite formed by the coupling of TiO_2_ with exposed high-energy facets and MoS_2_ microsphere. For example, Zhang et al. prepared a MoS_2_/TiO_2_(001) composite using the two-dimension MoS_2_ and anatase TiO_2_ with exposed {001} facets, which showed a large enhancement of photocatalytic activity for the degradation of MB [16]. Wei et al. prepared MoS_2_/{001}-TiO_2_ and MoS_2_{101}-TiO_2_, which showed that for {001} facets of TiO_2_ it was easy to accept electrons from MoS_2_, while {001} facets of TiO_2_ were conductive to the Z-scheme recombination of electrons in TiO_2_ with holes in MoS_2_, respectively [7].

In the present work, pH*x*-TiO_2_/MoS_2_ composites were synthesized via an effective hydrothermal route under different pH conditions. The synthesized composites were composed of anatase TiO_2_ and exposed with {101}/[111]-facets (or {101}/{001}/{010} facets) and MoS_2_ microsphere constructed by layer-by-layer self-assembly of nanosheets. The structure, morphology, microstructure, chemical composition, and optical properties of the pH*x*-TiO_2_/MoS_2_ composites were carefully investigated. The photocatalytic activity for the degradation of RhB under ultraviolet light irradiation was also investigated, and compared with that of CM-TiO_2_ and MoS_2_. The novel TiO_2_/MoS_2_ composites constructed by layer-by-layer self-assembly of nanosheets have shown higher ultraviolet light activity as well as higher stability. Our as-synthesized TiO_2_/MoS_2_ composites hold great potential for the future synthesis of effective composite photocatalysts.

## 2. Materials and Methods

### 2.1. Materials

Anhydrous sodium carbonate (Na_2_CO_3_, 99.0%), titanium dioxide (TiO_2_, 98.5%), nitric acid (HNO_3_, 65–68%), and tetramethylammonium hydroxide (TMAOH, 96%), sodium molybdate dehydrate (Na_2_MoO_4_·2H_2_O, 99.0%), and thiourea (CS(NH_2_)_2_, 99.0%) were purchased from Tianjin Hengxing Chemical Reagent Manufacturing Co., Ltd. (Tianjin, China), Tianjin Bodi Chemical Co., Ltd. (Tianjin, China), Damao Chemical Reagent Factory (Tianjin, China), Dibo Chemicals Technology Co., Ltd. (Shanghai, China), Bejing Chemical works (Beijing, China), and Tianjin Datum Chemical Reagent Co., Ltd. (Tianjin, China), respectively. The above chemical reagents were used as received without further purification.

### 2.2. Synthesis of MoS_2_ Crystal

A total of 4.00 g Na_2_MoO_4_·2H_2_O and 8.00 g CS(NH_2_)_2_ were dissolved in a 500 mL quartz beaker containing 240 mL of deionized water under continuous stirring for 30 min to form a homogeneous solution. The above mixture was divided into four equal parts and transferred to four teflon-lined stainless steel autoclaves with a volume of 100 mL. The autoclaves were incubated in a constant temperature blast drying oven at 180 °C for 24 h. After cooling to room temperature, the black precipitates were separated by filtration, dried at room temperature for 24 h, and finally calcined at 800 °C for 2 h under argon.

### 2.3. Synthesis of pHx-TiO_2_/MoS_2_ Composites

A total of 11.1290 g Na_2_CO_3_ and 23.97 g TiO_2_ were ground evenly in an agate mortar, transferred to a corundum crucible, and then calcined in a high-temperature box furnace at 900℃ for 24 h to prepare layered Na_2_Ti_3_O_7_. H_2_Ti_3_O_7_ was obtained through three cycles of ion exchange of 20.0 g Na_2_Ti_3_O_7_ in 2.0 L 1mol/L HNO_3_ aqueous solution. A total of 13.3623 g H_2_Ti_3_O_7_ and 16.9213 g TMAOH were dissolved in 130 mL deionized water, stirring continuously for 30 min, and then transferred equally into two 100 mL teflon-lined autoclaves. The above autoclaves were fixed in a homogeneous reactor and gently heated to 85 °C under air while stirring for 24 h. The resulting H_2_Ti_3_O_7_ colloidal suspension was redispersed in 400 mL deionized water while continuously stirring to ensure stabilization. An amount of 70 mL H_2_Ti_3_O_7_ colloidal suspension and 0.25 g MoS_2_ crystal prepared above were added to a 100 mL teflon-lined autoclave, and then adjusted to the set pH value (pH = 1.5, 3.5, 5.5, 7.5, 9.5, 11.5, and 12.5) under stirring conditions. The autoclaves were heated at 180 °C for 24 h in a constant temperature blast drying oven. The resulting gray and black powders were filtered and cleaned several times with distilled water. The final products pH*x*-TiO_2_/MoS_2_ (*x* is the pH of colloidal suspension, *x* = 1.5, 3.5, 5.5, 7.5, 9.5, 11.5, and 12.5, respectively) were obtained by drying at room temperature for longer than 24 h.

### 2.4. Sample Characterization

The Powder X-ray diffraction (XRD) patterns obtained on an X-ray diffractometer (XRD-6100, Shimadzu, Kyoto, Japan) using Cu Kα radiation (λ = 0.15406 nm) at a scanning speed of 5°/min were used to determine the crystal structure of the samples. The accelerating voltage and the applied current used in the measurement were 40.0 kV and 30.0 mA, respectively. The morphology of the precursor Na_2_Ti_3_O_7_, H_2_Ti_3_O_7_, MoS_2_, and the synthesized pH*x*-TiO_2_/MoS_2_ samples were observed using field emission scanning electron microscopy (FESEM, Hitachi SU8100, Tokyo, Japan). The microstructure details of the synthesized pH*x*-TiO_2_/MoS_2_ composites were investigated using transmission electron microscopy (TEM) and high-resolution transmission electron microscopy (HRTEM) (FEI TALO F200S at 200 kV, Portland, OR, USA). The surface chemical information of the samples was determined by X-ray photoelectron spectroscopy (XPS, Thermo Fisher Scientific, New York, NY, USA), and the binding energies were calibrated by the C 1s peak of extraneous carbon at 284.6 eV. The specific surface area was measured with a micromeritics ASAP 2020 nitrogen adsorption instrument (Micromeritics Instrument Corp., Atlanta, GA, USA) at 77 K using the Brunauer–Emmett–Teller (BET) equation. The photoluminescence (PL) spectra of the pH*x*-TiO_2_/MoS_2_ composites were recorded on a fluorescence spectrometer (HORIBA Fluoromax-4, HORIBA Instruments Inc., Kyoto, Japan) at room temperature and the emission spectrum was excited at a wavelength of 325 nm. Ultraviolet–visible (UV–Vis) absorption spectra of the samples were recorded within the 200~800 nm wavelength range on a UV–Vis spectrophotometer (UV-2600, Shimadzu, Kyoto, Japan).

### 2.5. Photocatalytic Experiments

Photocatalytic activity of the synthesized pH*x*-TiO_2_/MoS_2_ composites was evaluated with rhodamine B (RhB) dye as a model water pollutant under ultraviolet light irradiation. A total of 100 mg of the sample was dispersed within a 150 mL RhB aqueous solution with a concentration of 2.22 × 10^−5^ mol/L in a 250 mL quartz beaker. Before the photocatalytic activity test, the suspension was kept in the dark for 60 min under continuous stirring to achieve the adsorption/desorption equilibrium of the RhB on the pH*x*-TiO_2_/MoS_2_ composite surface. One 175 W low-pressure mercury lamp with a maximum emission at 365 nm was used as the ultraviolet light source, and the distance from the light source to the liquid surface of the suspension was 25 cm. At given time intervals, 4 mL of the suspension was extracted and analyzed after removal of solid particles by centrifugation at 1200 rpm for 5 min. The absorbance spectra of the supernatant liquors were recorded using an ultraviolet-visible spectrophotometer (TU 1901, Beijing Purkinje General Instrument Co., Ltd., Beijing, China). The photocatalytic activity of CM-TiO_2_ and MoS_2_ crystals was also measured as a reference to compare with that of the synthesized pH*x*-TiO_2_/MoS_2_ composites using the same parameters. The dye degradation efficiency was calculated according to the following formula [17]: Degradation rate (%) = (*c*_0_ − *c*_t_)/*c*_t_ × 100%, where *c*_0_ and *c*_t_ are the original RhB concentration after adsorption/desorption equilibrium and the residual RhB concentration after a certain period of illumination, respectively. 

## 3. Results and Discussion

### 3.1. XRD Analysis

**Figure 1:**
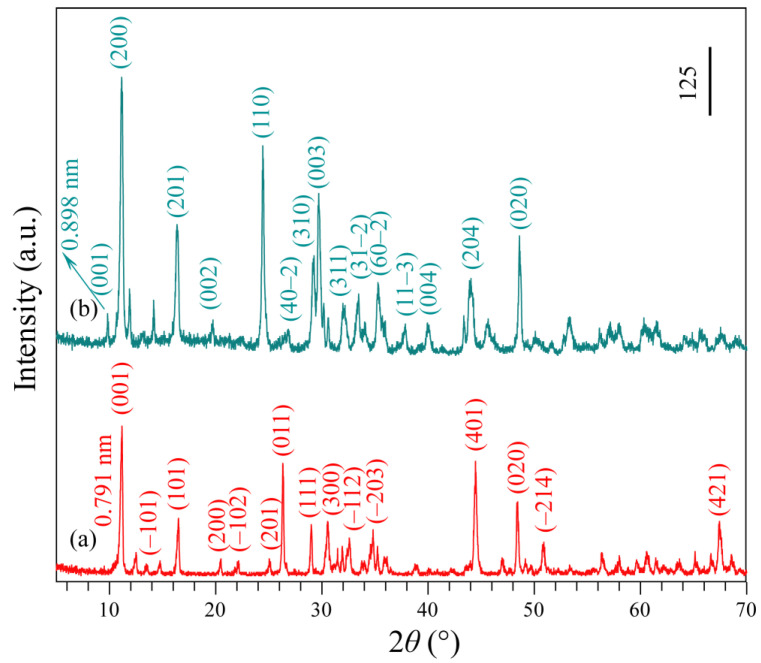
XRD patterns of (**a**) Na_2_Ti_3_O_7_ and (**b**) H_2_Ti_3_O_7_ samples.

Na_2_Ti_3_O_7_ was synthesized according to the literature by mixing TiO_2_ with a 5% stoichiometric excess of Na_2_CO_3_ uniformly and heating to 900 °C for 24 h. Then, the resulting Na_2_Ti_3_O_7_ white powder was reacted with 1 mol/L HNO_3_ aqueous solution for three days to convert it into H_2_Ti_3_O_7_. The crystallographic structure of the obtained Na_2_Ti_3_O_7_ and H_2_Ti_3_O_7_ samples were identified by XRD analysis. Figure 1a,b show the XRD patterns of the as-synthesized Na_2_Ti_3_O_7_ and H_2_Ti_3_O_7_ powders. The peaks observed at 11.18°, 13.56°, 16.50°, 20.49°, 22.16°, 25.06°, 26.32°, 29.00°, 30.54°, 32.58°, 34.82°, 44.46°, 48.44°, 50.90°, and 67.44°, can be readily indexed to the (001), (–101), (101), (200), (–102), (201), (011), (111), (300), (–112), (–203), (401), (020), (–214), and (421) planes of monoclinic Na_2_Ti_3_O_7_ layered structure with lattice constants *a* = 9.128 Å, *b* = 3.803 Å, and *c* = 8.562 Å (JCPDS file no. 31-1329). The peaks located at 9.84°, 11.14°, 16.36°, 19.74°, 24.44°, 26.84°,29.26°, 29.70°, 32.08°, 33.46°, 35.28°, 37.86°, 40.08°, 43.90°, and 48.60° are indexed to (001), (200), (201), (002), (110), (40–2), (310), (003), (311), (31–2), (60–2), (11–3), (004), (204), and (020) diffraction peaks of layered H_2_Ti_3_O_7_ (monoclinic structure, JCPDS file no. 41-0192, *a* = 15.99 Å, *b* = 3.783 Å, and *c* = 9.172 Å). After the Na^+^/H^+^ ion-exchange reaction, the sample retained the monoclinic layered structure, and the basal spacing of (001) plane was changed from 0.791 nm (Figure 1a) to 0.898 nm (Figure 1b), indicating that H_3_O^+^ and H_2_O exist in the interlayer space of the obtained H^+^-form titanate H_2_Ti_3_O_7_ [18].

**Figure 2:**
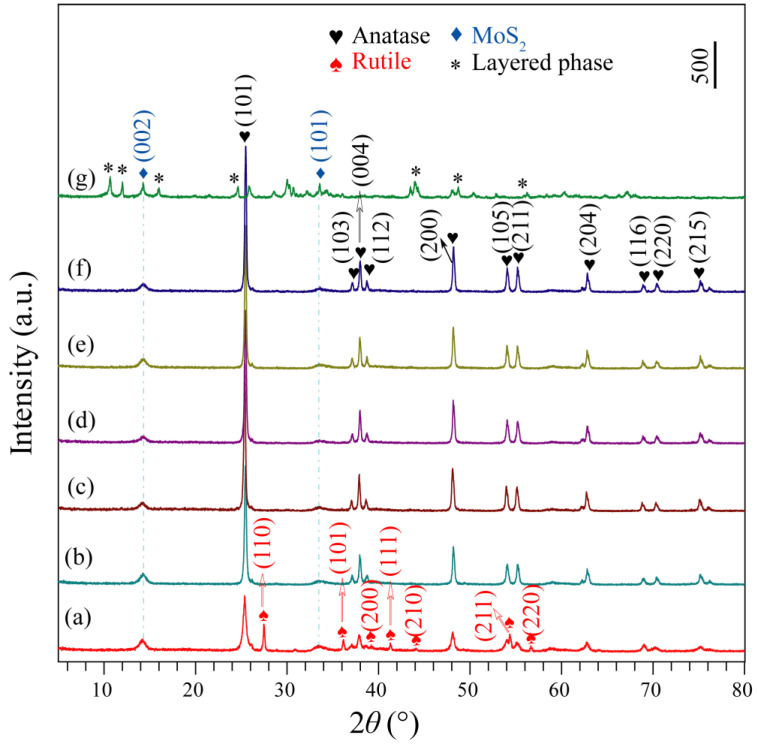
XRD patterns of (**a**) pH1.5-TiO_2_/MoS_2_, (**b**) pH3.5-TiO_2_/MoS_2_, (**c**) pH5.5-TiO_2_/MoS_2_, (**d**) pH7.5-TiO_2_/MoS_2_, (**e**) pH9.5-TiO_2_/MoS_2_, (**f**) pH11.5-TiO_2_/MoS_2_, and (**g**) pH12.5-TiO_2_/MoS_2_ composites synthesized under different pH conditions.

Figure 2 shows the XRD patterns of the TiO_2_/MoS_2_ composites prepared under different pH conditions. Figure 2a shows a typical XRD pattern of the pH1.5-TiO_2_/MoS_2_ composite, which clearly demonstrates the coexistence of three crystal structures, namely hexagonal MoS_2_ (2H phase, JCPDS file no. 37-1492: *a* = *b* = 3.161 Å, *c* =12.299 Å; spacing group: *P*6_3_/*mmc*), tetragonal anatase (JCPDS file no. 21-1272: *a* = *b* = 3.785 Å, *c* = 9.514 Å; spacing group: *I*4_1_/*amd*) and tetragonal rutile (JCPDS file no. 21-1276: *a* = *b* = 4.593 Å, *c* = 2.959 Å; spacing group: *P*4_2_/*mmm*). The diffraction peaks observed at 14.26° and 36.16° are in good agreement with the (002) and (101) crystal planes of MoS_2_. The diffraction peaks at 2*θ* = 25.36°, 37.08°, 37.88°, 38.74°, 48.12°, 54.01°, 55.14°, 62.82°, 69.04°, 70.36°, and 75.38° are indexed to (101), (103), (004), (112), (200), (105), (211), (204), (116), (220), and (215) planes of monoclinic anatase TiO_2_. The diffraction peaks at 2*θ* = 27.46°, 36.16°, 39.21°, 41.28°, 44.08°, 54.34°, and 56.79° correspond to the (100), (101), (200), (111), (210), (211), and (220) planes, which match well with the standard peaks of monoclinic rutile TiO_2_. It can be clearly seen from Figure 2b–g that the pH3.5-TiO_2_/MoS_2_, pH5.5-TiO_2_/MoS_2_, pH7.5-TiO_2_/MoS_2_, pH9.5-TiO_2_/MoS_2_, and pH11.5-TiO_2_/MoS_2_ composites exhibit an anatase TiO_2_ and 2H-MoS_2_ coexisting diffraction pattern, while pH12.5-TiO_2_/MoS_2_ composite shows a MoS_2_/layered phase coexisting diffraction pattern. Aside from pH12.5-TiO_2_/MoS_2_ composite, all of the as-prepared TiO_2_/MoS_2_ composites have obvious strong diffraction peaks, implying the good crystallinity [19], and with increasing the pH values, the peak intensity of anatase TiO_2_ (101) plane steadily increases and the width becomes narrower, indicating that the crystallinity of anatase TiO_2_ in the TiO_2_/MoS_2_ composites is improved [20].

### 3.2. Morphology and Microstructure Analysis

**Figure 3:**
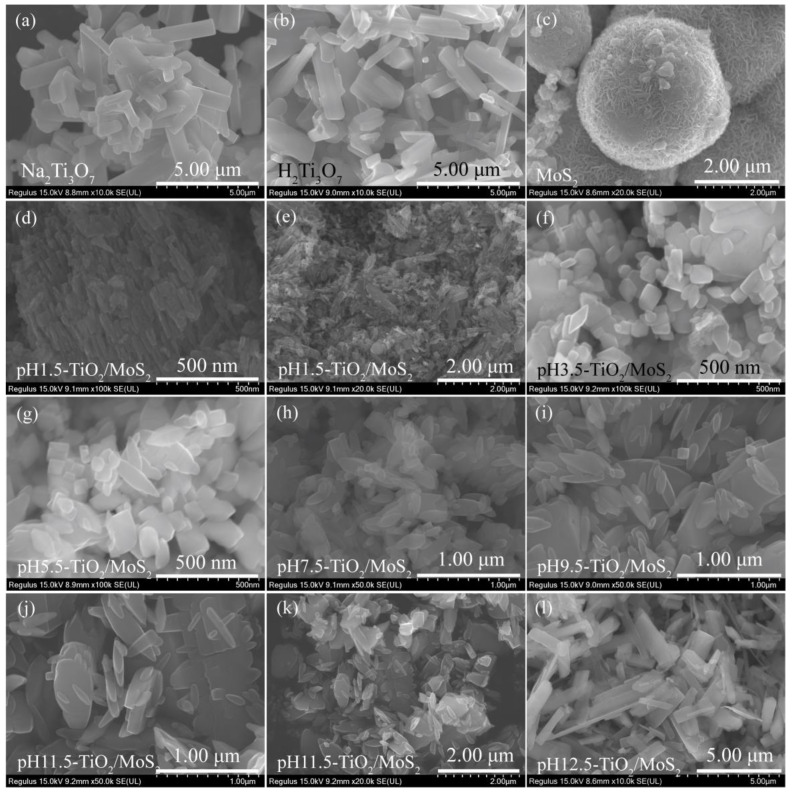
FESEM images of (**a**) Na_2_Ti_3_O_7_, (**b**) H_2_Ti_3_O_7_, (**c**) MoS_2_, (**d**,**e**) pH1.5-TiO_2_/MoS_2_, (**f**) pH3.5-TiO_2_/MoS_2_, (**g**) pH5.5-TiO_2_/MoS_2_, (**h**) pH7.5-TiO_2_/MoS_2_, (**i**) pH9.5-TiO_2_/MoS_2_, (**j**,**k**) pH11.5-TiO_2_/MoS_2_, and (**l**) pH12.5-TiO_2_/MoS_2_ samples.

Figure 3a,b show the FESEM images of the as-prepared Na_2_Ti_3_O_7_ and H_2_Ti_3_O_7_ samples. It can be seen that the morphology of H_2_Ti_3_O_7_ obtained after proton exchange is consistent with that of the precursor Na_2_Ti_3_O_7_, both of which are tetragonal microrods with a length of *ca.* 0.8–3.6 μm, a width of *ca.* 0.18–1.0 μm, and a thickness of *ca.* 0.09–0.68 μm. As shown in Figure 3c, the morphology of MoS_2_ crystal prepared by hydrothermal method is microspheres with a diameter of *ca.* 1.76–4.05 μm constructed by layer-by-layer self-assembly of nanosheets with a thickness of 5.1–11.9 nm. Figure 3d–l shows the FESEM images of the pH*x*-TiO_2_/MoS_2_ composites obtained by hydrothermal treatment of the colloidal suspension of H_2_Ti_3_O_7_ containing MoS_2_ microspheres under different pH conditions. No MoS_2_ microspheres were observed in the SEM images of pH*x*-TiO_2_/MoS_2_ composites, which may be caused by the low content of MoS_2_ and the small observed area. In addition, it can be seen from Figure 3d–l that the pH of the colloidal suspension of H_2_Ti_3_O_7_ has an important influence on the morphology of the synthesized pH*x*-TiO_2_/MoS_2_ composites. When the pH is 1.5, the morphology of the obtained pH1.5-TiO_2_/MoS_2_ composite is mainly rod-shaped with a length of 21.5–188.5 nm and a width of 9.5–34.5 nm, corresponding to anatase TiO_2_ (Figure 3d). In addition, some square rod-shaped large particles with a length of 0.49–1.17 μm and a width of 0.16–0.23 μm were also observed, corresponding to rutile TiO_2_ (Figure 3e). When the pH is 3.5, the morphology of obtained pH3.5-TiO_2_/MoS_2_ composite is mainly composed of rhomboids with a length of 80–180 nm and a width of 37.5–78.0 nm, and cuboids with a length of 43–170 nm and a width of 34.5–108 nm (Figure 3f). When the pH is 5.5, the morphology of obtained pH5.5-TiO_2_/MoS_2_ composite is mainly composed of spindles with a length of 0.16–0.60 μm and a width of 60–160 nm, and cuboids with a length of 56–186 nm and a width of 40–110 nm (Figure 3g). When the pH is 7.5, the morphology of the obtained pH7.5-TiO_2_/MoS_2_ composite is mainly spindle-shaped with a length of 0.09–0.62 μm and a width of 0.04–0.16 μm. In addition, a few cuboids with a length of 0.10–0.24 μm a width of 0.06–0.17 μm, and irregular large particles are also observed (Figure 3h). When the pH is 9.5 and 11.5, the morphology of the pH9.5-TiO_2_/MoS_2_ and pH11.5-TiO_2_/MoS_2_ composites are mainly spindle-shaped and irregular, and the spindle was obtained by cracking the tetragonal microrods-shaped H_2_Ti_3_O_7_ along a certain direction (Figure 3i–k). However, when the pH continues to increase to 12.5, the morphology of the obtained pH12.5-TiO_2_/MoS_2_ composite is consistent with that of the H_2_Ti_3_O_7_, which is tetragonal microrods-shaped morphology (Figure 3l). Combined with the above XRD analysis, it can be seen that the morphology is the unreacted layered phase.

**Figure 4:**
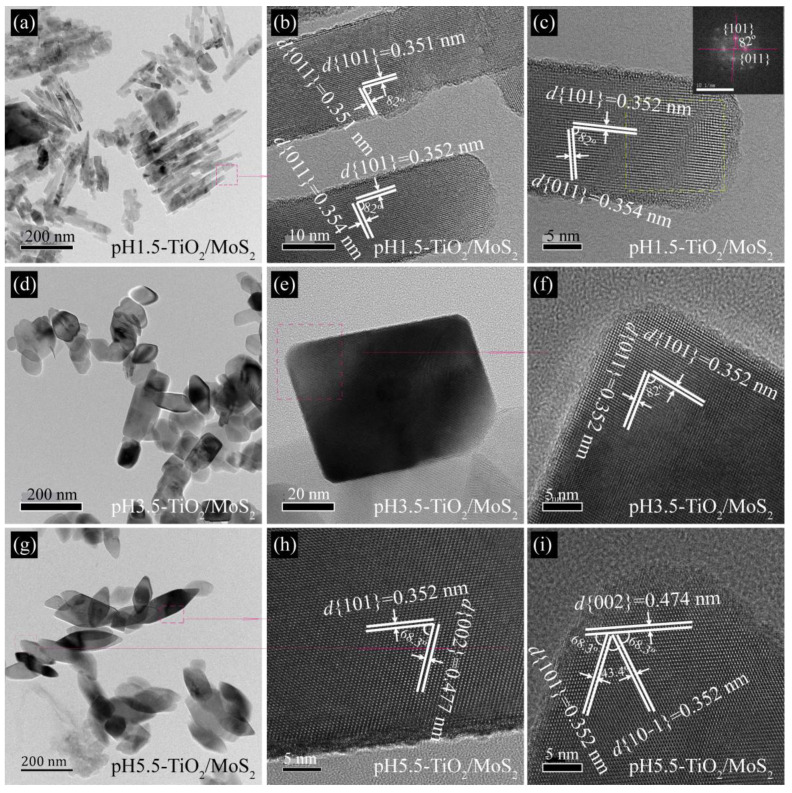
Representative TEM and HRTEM images of (**a**–**c**) pH1.5-TiO_2_/MoS_2_, (**d**–**f**) pH3.5-TiO_2_/MoS_2_, and (**g**–**i**) pH5.5-TiO_2_/MoS_2_ composites.

**Figure 5:**
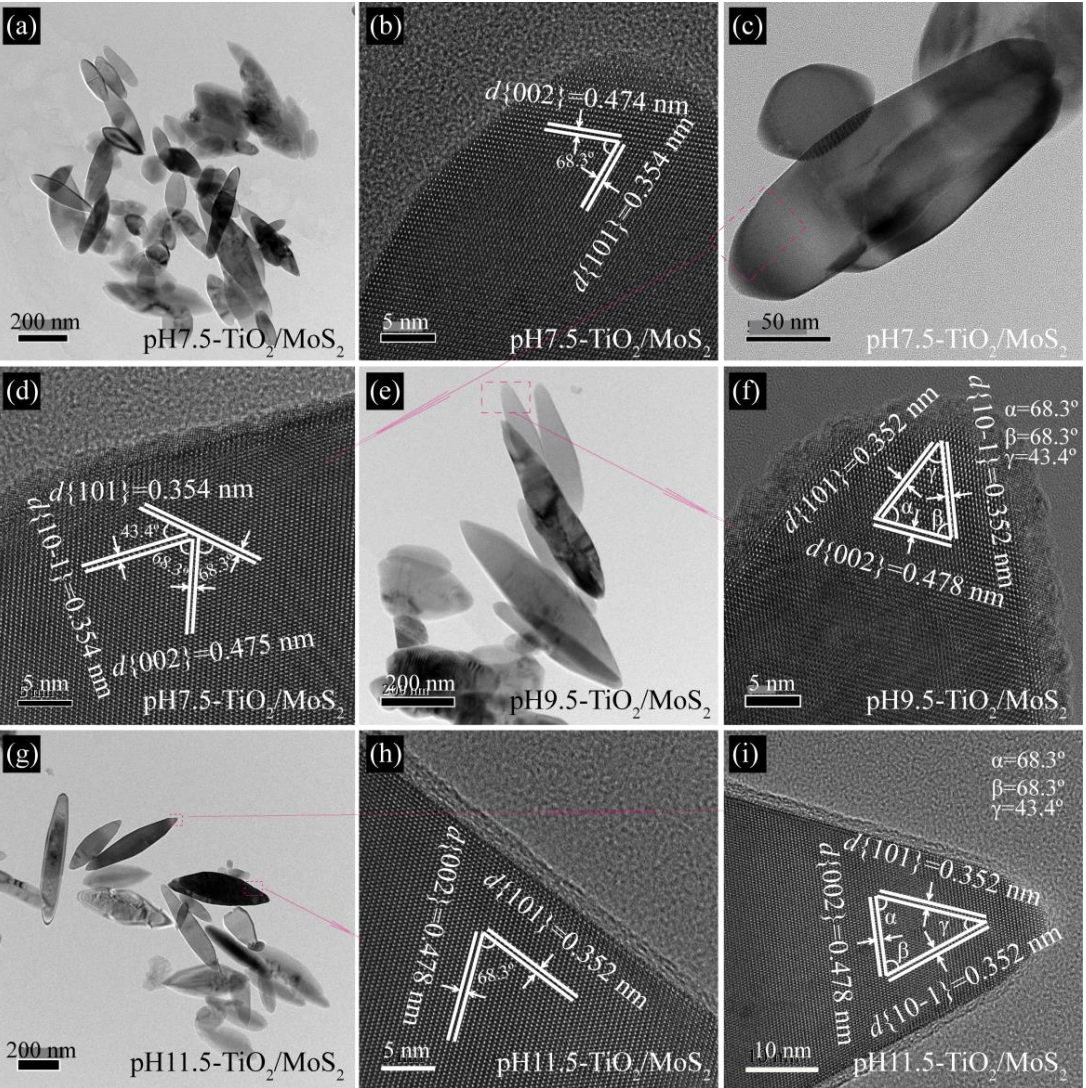
Representative TEM and HRTEM images of (**a**–**c**) pH7.5-TiO_2_/MoS_2_, (**d**–**f**) pH9.5-TiO_2_/MoS_2_, and (**g**–**i**) pH11.5-TiO_2_/MoS_2_ composites.

The microstructures of pH*x*-TiO_2_/MoS_2_ composites are confirmed further by TEM and HRTEM images. The TEM images (Figure 4 and Figure 5) show the similar morphology to the FESEM images. As shown in Figure 4a, the nanorods in the obtained pH1.5-TiO_2_/MoS_2_ composite have a length of 20.0–152.5 nm and a width of 10.0–21.4 nm, which are in agreement with the SEM image. The nanorod structure of the pH1.5-TiO_2_/MoS_2_ composite can be also confirmed by HRTEM. From HRTEM images (Figure 4b,c) the lattice spacing parallel to the top and side facets of the nanorod was determined to be 0.351 nm (or 0.354 nm) and 0.351 nm (or 0.352 nm), which corresponds to the (011) and (101) facets, respectively. Additionally, the interfacial angle between the (011) and (101) crystal planes is 82°, which is consistent with the theoretical value, indicating that the co-exposed crystal planes of the nanorods are {101} facets and the crystal planes perpendicular to the [111]-crystal axis (denoted as [111]-facets). The fast fourier transform (FFT) diffraction pattern of the yellow, dashed line region (Figure 4c inset) further indicates that the anatase TiO_2_ nanocrystal is a single-crystalline and co-exposed crystal planes are {101} and [111]-facets. Figure 4d shows a typical TEM image of pH3.5-TiO_2_/MoS_2_ composite obtained, revealing that the pH3.5-TiO_2_/MoS_2_ composite are composed of rhomboids with a length of 84–135 nm and a width of about 60 nm, and cuboids with a length of 36–164 nm and a width of 35–79 nm, consistent with FESEM observations. Figure 4e shows a TEM image of an individual cuboid with a size of about 72.3 × 55.2 nm. Two sets of lattice fringes with interplanar spacings of 0.352 nm and an interfacial angle of 82° are observed, indicating that the co-exposed crystal panes of cuboid are also {101} and [111]-facets (Figure 4f). The pH5.5-TiO_2_/MoS_2_ composite is mainly composed of spindles with a length of 178–268 nm and a width of 38–77 nm and cuboids with a length of 58–130 nm and a width of 50–81 nm, as shown in Figure 4g. The lattice fringes with spacing of 0.352 and 0.447 nm can be ascribed to the (101) and (002) crystal planes of anatase TiO_2_, and the interfacial angle of 68.3° is identical to the theoretical value for the angle between the (101) and (002) crystal planes of anatase TiO_2_, indicating the preferred growth direction of the spindle along the *c*-axis and the co-exposed crystal planes are {010} and {101} facets (Figure 4h) [21]. The three sets of lattice fringes with interplanar spacings of 0.352, 0.352 and 0.474 nm can be assigned to the (101), (10−1) and (002) planes of anatase TiO_2_, respectively (Figure 4i). Three interfacial angles of 43.4°, 68.3° and 68.3° observed on the spindle top are consistent with the theoretical values for the angles between (101) and (10−1), (101) and (002), and (10−1) and (101) planes. In addition, it is observed that the (101) and (10−1) planes are parallel to two sides of the spindle crystal. The above analysis shows that the co-exposed crystal planes of spindles are {101} and {010} facets.

Figure 5a shows a typical TEM image of pH7.5-TiO_2_/MoS_2_ composite obtained, revealing that the pH7.5-TiO_2_/MoS_2_ composite are composed of spindles with a length of 130–444 nm and a width of 42–135 nm, consistent with FESEM observations. The lattice fringes parallel to the side and top of the spindle with spacings of 0.354 and 0.474 nm can be ascribed to (101) and (002) planes of anatase TiO_2_, respectively, and the interfacial angle of 68.3° is consistent with the theoretical values for the angle between (101) and (002) planes, indicating that the co-exposed crystal planes of the spindle are {101}/{010} facets (Figure 5b). Figure 5d is a HRTEM image of the pink dotted rectangle in Figure 5c. The three sets of lattice fringes with interplanar spacings of 0.354, 0.354 and 0.475 nm can be assigned to the (101), (10−1) and (002) planes of anatase TiO_2_, respectively (Figure 5d). Three interfacial angles of 43.4°, 68.3° and 68.3° observed on the spindle top are consistent with the theoretical values for the angles between (101) and (10−1), (101) and (002), and (10−1) and (101) planes. The above analysis shows that the co-exposed crystal planes of spindles are {101} and {010} facets. When the pH increased to 9.5 and 11.5, the obtained pH9.5-TiO_2_/MoS_2_ and pH11.5-TiO_2_/MoS_2_ composites still remained the spindle morphology, which was consistent with the FESEM results (Figure 5e,g). The corresponding HRTEM images (Figure 5f,h,i) further confirmed that the spindles co-exposed {101} and {010} facets.

### 3.3. XPS Studies

**Figure 6:**
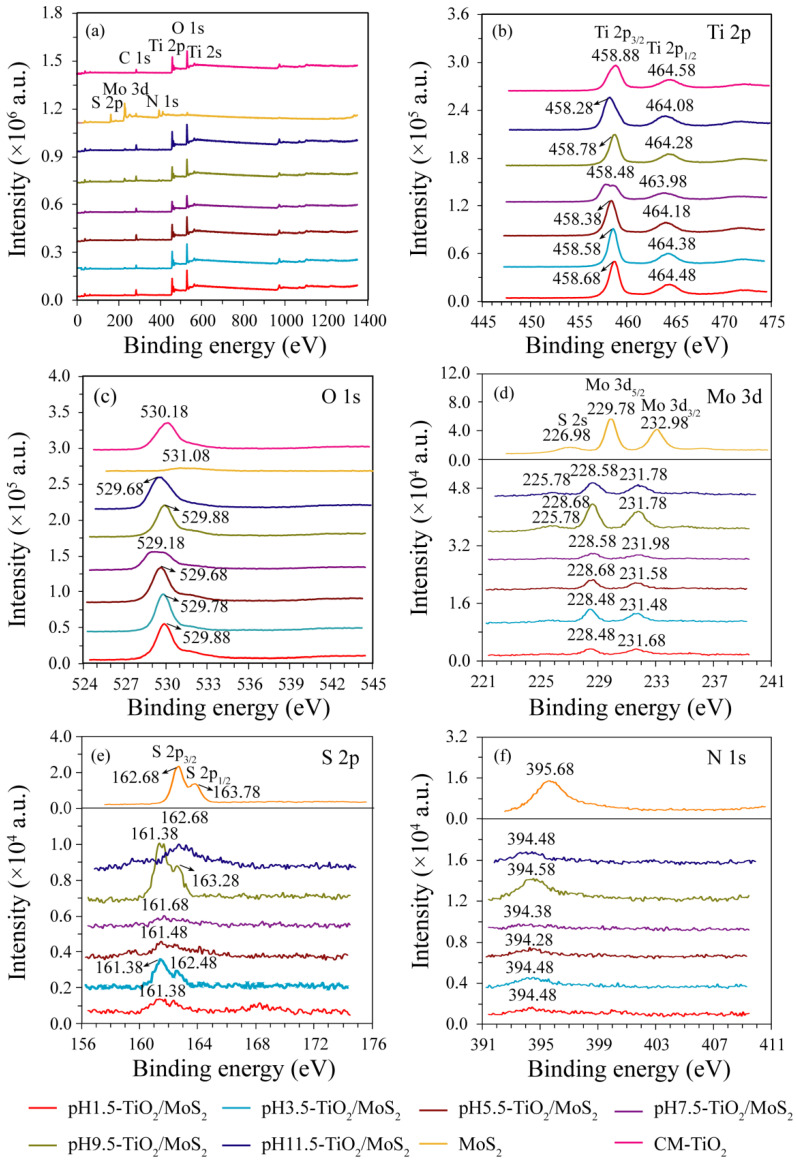
XPS survey spectra of (**a**) TiO_2_/MoS_2_ composites, MoS_2_ and CM-TiO_2_ crystals, and high-resolution spectra of (**b**) Ti 2p, (**c**) O 1s, (**d**) Mo 3d, (**e**) S 2p, and (**f**) N 1s.

The chemical composition and purity of the prepared samples were analyzed by X-ray photoelectron spectroscopy (XPS). Figure 6 shows XPS survey spectra of the pH*x*-TiO_2_/MoS_2_ composites, MoS_2_ and CM-TiO_2_ crystals, and the high-resolution spectra of Ti 2p, O 1s, Mo 3d, S 2p, and N 1s. It can be seen from Figure 6a that all the pH*x*-TiO_2_/MoS_2_ composites contain not only Ti, O, Mo, and S elements, but also C elements, with sharp photoelectron peaks appearing at binding energies of 564 (Ti 2s), 529 (O 1s), 458 (Ti 2p), 284 (C 1s), 228 (Mo 3d), and 162 eV (S 2p). The XPS survey spectrum also reveals that the sample MoS_2_ (or CM-TiO_2_) contains five elements, O, N, C, Mo, S (or three elements Ti, O, C), in which the chemical binding energies of O 1s, N1s, C 1s, Mo 3d, and S 2p (or Ti2s, O 1s, Ti 2p and C 1s) are 531, 395, 284, 229, and 162 eV (or 566, 530, 459, and 285 eV), respectively, as shown in Figure 6a. The C 1s peak at 284 eV (or 285 eV) is the signal from residual carbon and the adventitious hydrocarbon of the XPS instrument itself [22]. The Ti 2p_3/2_ and Ti 2p_1/2_ peaks are observed from the high-resolution spectra of Ti 2p at binding energies of 458.28~458.88 eV and 463.98~464.58 eV, respectively, demonstrating the Ti^4+^ nature of the titanium in the as-prepared pH*x*-TiO_2_/MoS_2_ composites and CM-TiO_2_ crystals, as shown in Figure 6b [23]. The single O 1s peak (Figure 6c) at 529.18~530.18 eV corresponding to Ti-O-Ti bonds present in the pH*x*-TiO_2_/MoS_2_ composites and CM-TiO_2_ crystals. The O 1s peak at 531.08 eV can be attributed to hydroxyl species generated by the adsorption of contaminants on the surface of MoS_2_ crystal [24]. The high resolution XPS spectra of the Mo 3d region (Figure 6d) show binding energy peaks at 228.48~229.78 eV for Mo 3d_5/2_ and at 231.48~232.98 eV for Mo 3d_3/2_, suggesting that Mo exists in the chemical state of Mo^4+^ in the pure MoS_2_ crystal and pH*x*-TiO_2_/MoS_2_ composites [25]. The S 2s peaks (Figure 6d) are also observed at 225.78 eV for pH9.5-TiO_2_/MoS_2_ (or pH11.5-TiO_2_/MoS_2_) and at 226.98 eV for pure MoS_2_. The S 2p_3/2_ and S 2p_1/2_ peaks (Figure 6e) are observed at 161.38~161.68 eV for pH*x*-TiO_2_/MoS_2_ (*x* = 1.5~9.5, or 162.68 eV for pH11.5-TiO_2_/MoS_2_ and pure MoS_2_) and at 162.48 eV for pH3.5-TiO_2_/MoS_2_ (or at 163.28 eV for pH9.5-TiO_2_/MoS_2_ or at 163.78 eV for pure MoS_2_), respectively, which confirmed that S element exists mainly in the form of S^2−^ on the pH*x*-TiO_2_/MoS_2_ composites and pure MoS_2_ crystal surface [26]. The peaks of the pH*x*-TiO_2_/MoS_2_ composites at 394.28~395.68 eV can be assigned to N 1s, which come from the raw material thiourea or tetrahydroxy ammonium hydroxide used in the synthesis process. Therefore, the sample pH*x*-TiO_2_/MoS_2_ is further confirmed to be TiO_2_/MoS_2_ composite, which is consistent with the XRD results. The positions of Ti 2p and Mo 3d peaks of pH*x*-TiO_2_/MoS_2_ composites are slightly shifted, which are due to the different atomic arrangements and configurations of the each dominant facet of the as-prepared TiO_2_/MoS_2_ composites, resulting in different surface electronic structures [27]. The discrepancy of peak intensities Ti 2p, O 1s, Mo 3d, S 2p, and N 1s of pH*x*-TiO_2_/MoS_2_ composites is caused by the difference in the content of Ti, O, Mo, S, and N elements.

### 3.4. Optical Studies

**Figure 7:**
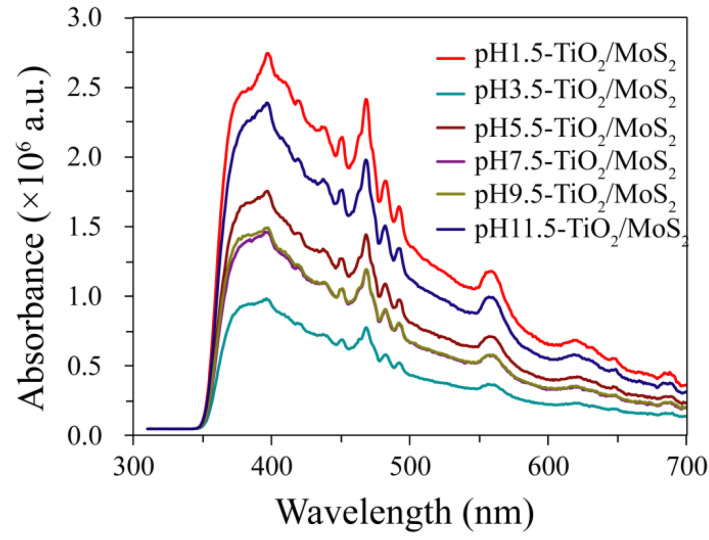
Photoluminescence spectra of the as-prepared TiO_2_/MoS_2_ composites.

The photo-electron and hole separation efficiency of different TiO_2_/MoS_2_ composites was investigated by photoluminescence (PL) emission spectroscopy. As shown in Figure 7, the PL spectra of the pH*x*-TiO_2_/MoS_2_ composites under excitation at 325 nm exhibit fluorescence emission in the range of 310 ~ 700 nm, which are caused by the recombination of photoexcited holes and electrons. It can be seen that the emission intensity of the pH*x*-TiO_2_/MoS_2_ composites decreases in the order of pH1.5-TiO_2_/MoS_2_ > pH11.5-TiO_2_/MoS_2_ > pH5.5-TiO_2_/MoS_2_ > pH7.5-TiO_2_/MoS_2_ ≈ pH9.5-TiO_2_/MoS_2_ > pH3.5-TiO_2_/MoS_2_. Among the six different pH*x*-TiO_2_/MoS_2_ composites, the pH1.5-TiO_2_/MoS_2_ composite shows the strongest emission intensity, implying the highest charge-carrier recombination rate [28]. By contrast, the pH3.5-TiO_2_/MoS_2_ composite exhibits the weakest emission intensity, indicating that it has the highest separation efficiency of the photo-generated electrons and holes, and the best photocatalytic activity [29].

**Figure 8:**
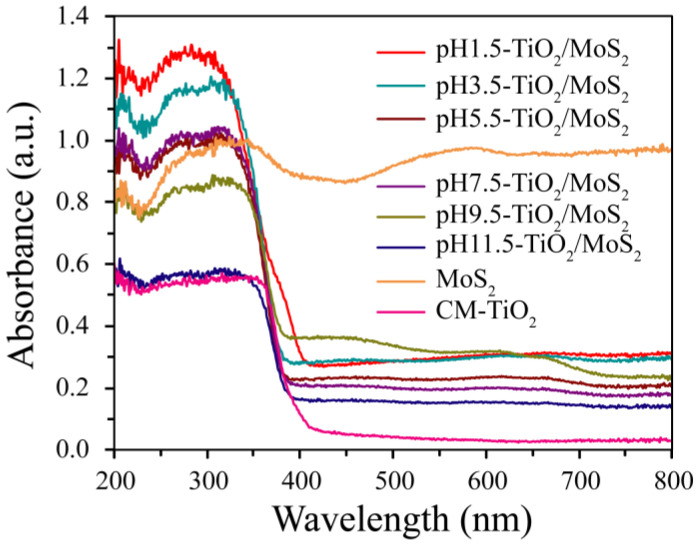
UV-visible absorption spectra of the as-prepared TiO_2_/MoS_2_ composites, CM-TiO_2_ crystals and MoS_2_ crystals.

The light absorption properties of pH*x*-TiO_2_/MoS_2_ composites, pure MoS_2_ and CM-TiO_2_ crystals were evaluated by UV-visible absorption spectra. It can be clearly seen from Figure 8 that all samples except MoS_2_ show strong absorption and steep absorption edges in the UV region, which is caused by the intrinsic bandgap absorption of TiO_2_ [30]. The absorption edges of pure CM-TiO_2_ and MoS_2_ were about 393 and 439 nm, while the absorption edges of pH1.5-TiO_2_/MoS_2_, pH3.5-TiO_2_/MoS_2_, pH5.5-TiO_2_/MoS_2_, pH7.5-TiO_2_/MoS_2_, pH9.5-TiO_2_/MoS_2_, pH11.5-TiO_2_/MoS_2_ composites show obvious blue shift of 2 and 48 nm (about 391 nm), 15 and 61 nm (about 378 nm), 14 and 60 nm (about 379 nm), 13 and 59 nm (about 380 nm), 13 and 59 nm (about 380 nm), 8 and 54 nm (about 385 nm) relative to that of pure CM-TiO_2_ and MoS_2_ crystals, respectively. According to the formula *E*_g_ = 1240/*λ* [31,32], where *λ* is wavelength of the absorption edge, the band gap (*E*_g_) values of 3.17, 3.28, 3.27, 3.26, 3.26, 3.22, 3.16, and 2.82 eV for 1.5-TiO_2_/MoS_2_, pH3.5-TiO_2_/MoS_2_, pH5.5-TiO_2_/MoS_2_, pH7.5-TiO_2_/MoS_2_, pH9.5-TiO_2_/MoS_2_, pH11.5-TiO_2_/MoS_2_, CM-TiO_2_ and MoS_2_ samples can be calculated, respectively. Furthermore, no absorption peaks appeared in the visible region of the pH*x*-TiO_2_/MoS_2_ composites, indicating that the deposited MoS_2_ has no energy gap at the fermi level [30].

### 3.5. Photocatalytic Response of TiO_2_/MoS_2_ Composites

**Figure 9:**
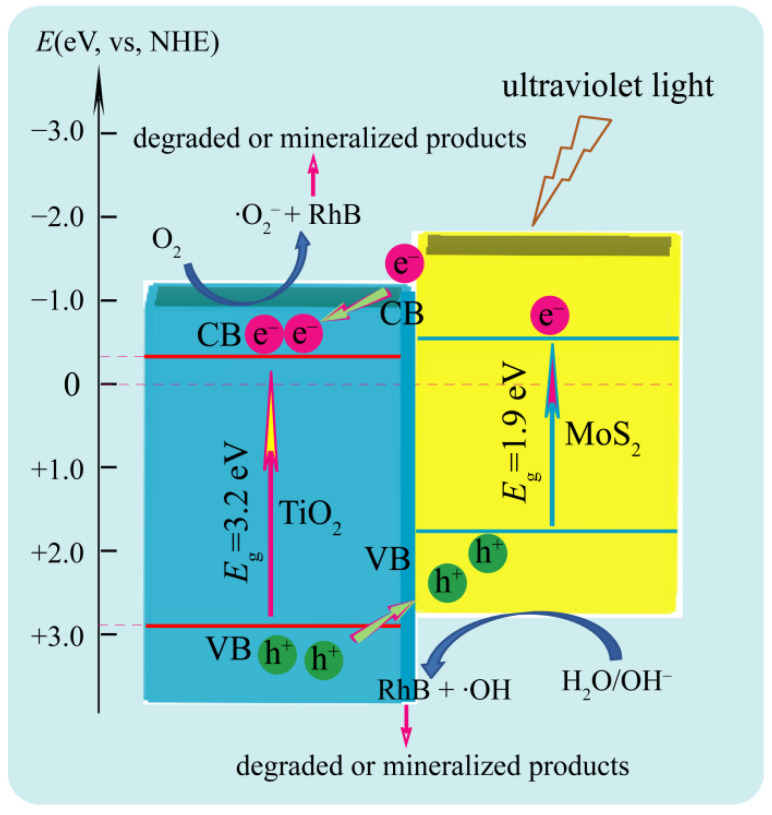
Possible photocatalytic mechanism of TiO2/MoS2 composites for the degradation of RhB under ultraviolet irradiation.

The photocatalytic activity of the as-prepared TiO_2_/MoS_2_ composites was measured using rhodamine B (RhB) as the target organic pollutant under ultraviolet light irradiation. A possible photocatalytic mechanism for the photogenerated electron-hole separation and transfer process between TiO_2_ nanocrystal and MoS_2_ crystal is shown in Figure 9. Under the ultraviolet light irradiation, the photogenerated electrons (e¯) were transferred from the conduction band (CB) of MoS_2_ to the CB of TiO_2_, and eventually reacted with the oxygen molecules absorbed on its surface to yield the superoxide radical anion (·O_2_¯). Simultaneously, the photogenerated holes (h^+^) were transferred from the valence band (VB) of TiO_2_ to the VB of MoS_2_, and eventually reacted with the surface water molecules and/or the surface OH^¯^ groups to yield the ·OH radicals [33,34]. RhB can be oxidized to peroxy or hydroxylated…intermediates by the active oxygen species O_2_¯ or·OH radicals, which is eventually degraded or mineralized [34]. The possible photocatalytic degradation mechanisms can be described as the following reaction:

TiO_2_ (e¯) + O_2_ →TiO_2_ (·O_2_¯)

TiO_2_ (·O_2_¯) + RhB → peroxy or hydroxylated…intermediates →→ degraded or mineralized products

MoS_2_ (h^+^) + H_2_O → MoS_2_ (·OH)

MoS_2_ (h^+^) + OH^¯^ → MoS_2_ (·OH)

MoS_2_ (·OH) + RhB → peroxy or hydroxylated…intermediates →→ degraded or mineralized products

**Figure 10:**
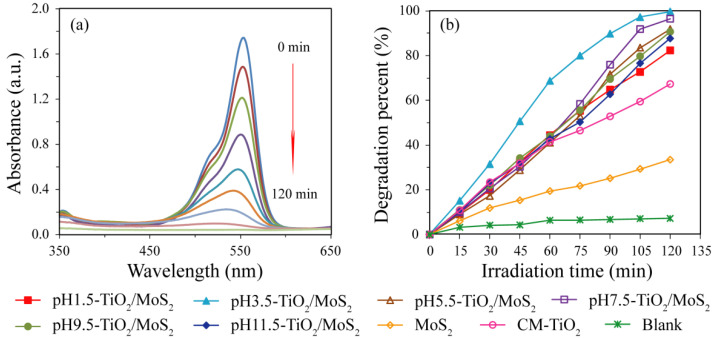
(**a**) spectral changes of the centrifuged rhodamine B solution with pH3.5-TiO_2_/MoS_2_ composite as the photocatalyst, and (**b**) Variation of degradation percentage of rhodamine B with irradiation time under ultraviolet light irradiation for pH*x*-TiO_2_/MoS_2_ composites, MoS_2_ crystals, CM-TiO_2_ crystals, and Blank.

Figure 10a shows the spectral changes of the centrifuged RhB solution with pH3.5-TiO_2_/MoS_2_ composite as the photocatalyst. It can be seen that the RhB shows a maximum absorption peak at 554 nm, and the intensity of the absorption peak gradually weakens with the prolonging of irradiation time, indicating that the RhB has been degraded. The degradation percentage of RhB with irradiation time for the as-prepared TiO_2_/MoS_2_ composites, MoS_2_ crystals, CM-TiO_2_ crystals and Blank are shown in Figure 10b, and the corresponding degradation rate values are given in Table 1. It is clear that pH3.5-TiO_2_/MoS_2_ composite shows higher photocatalytic activity than the other five TiO_2_/MoS_2_ composites. From the degradation percentage, it can be observed that the photocatalytic activity decreases in the order of pH3.5-TiO_2_/MoS_2_ (99.70%) > pH7.5-TiO_2_/MoS_2_ (96.46%) > pH5.5-TiO_2_/MoS_2_ (91.86%) > pH9.5-TiO_2_/MoS_2_ (90.68%) > pH11.5-TiO_2_/MoS_2_ (87.67%) > pH1.5-TiO_2_/MoS_2_ (82.33%) > CM-TiO_2_ (67.31%) > MoS_2_ (33.47%) > Blank (8.87%). The six TiO_2_/MoS_2_ composites synthesized under different pH conditions have different phase structure, particle size, specific surface area, and exposed crystal facets. It is reported that among the three common crystalline phases (anatase, rutile, and brookite) of TiO_2_, anatase TiO_2_ possesses the higher photocatalytic activity in photocatalytic degradation of organic pollutants [32,35,36]. The difference in photocatalytic activity of the synthesized TiO_2_/MoS_2_ composites is related to their phase structure, particle size, specific surface area, and exposed crystals facets. Generally speaking, the specific surface area of solid materials increases with the decrease in crystalline size, and particle surface with larger specific surface area can provide more active sites and adsorb more reactive species, thus promoting the improvement of photocatalytic activity [37]. Although the specific surface area (29.87 g/m^2^) of pH1.5-TiO_2_/MoS_2_ in the synthesized TiO_2_/MoS_2_ composites is the largest, the TiO_2_ in the composite is a mixed phase of anatase TiO_2_ (64.39%) and rutile TiO_2_ (35.61%), while the TiO_2_ in other TiO_2_/MoS_2_ composites are all single anatase phase, so pH1.5-TiO_2_/MoS_2_ composite exhibits the lowest photocatalytic activity. For other TiO_2_/MoS_2_ composites composed of single anatase TiO_2_, the order of increasing photocatalytic activity (pH11.5-TiO_2_/MoS_2_ (87.67%) < pH9.5-TiO_2_/MoS_2_ (90.68%) < pH5.5-TiO_2_/MoS_2_ (91.86%) < pH7.5-TiO_2_/MoS_2_ (96.46%) < pH3.5-TiO_2_/MoS_2_ (99.70%)) is exactly the same as that of increasing specific surface area (pH11.5-TiO_2_/MoS_2_ (5.74 g/m^2^) < pH9.5-TiO_2_/MoS_2_ (7.48 g/m^2^) < pH5.5-TiO_2_/MoS_2_ (9.93 g/m^2^) < pH7.5-TiO_2_/MoS_2_ (10.05 g/m^2^) < pH3.5- TiO_2_/MoS_2_ (11.08 g/m^2^)). The photocatalytic activity of CM-TiO_2_ crystals with larger specific surface area (25.61 m^2^/g) is lower than that of the as-prepared TiO_2_/MoS_2_ composites, indicating that the heterojunction structure formed between TiO_2_ and MoS_2_ contributes to the improvement of photocatalytic activity. The degradation percent of RhB by MoS_2_ is far lower than that of CM-TiO_2_ and TiO_2_/MoS_2_ composites, which may be caused by its small specific surface area (4.43 m^2^/g). Based on the above analysis, the pH3.5-TiO_2_/MoS_2_ composite has the highest photocatalytic activity which can be attributed to the cooperated effects of the smallest crystalline size, largest specific surface area, suitable heterojunction structure between anatase TiO_2_ and MoS_2_, and lowest fluorescence intensity.

To characterize the surface photocatalytic activity of TiO_2_/MoS_2_ composites, MoS_2_ crystals, and CM-TiO_2_ crystals, we evaluated the degradation amount of RhB per specific surface area of the composites. The degradation amount at 120 min were 0.37, 1.07, 1.29, 1.31, 1.59, 2.17, 1.04, and 0.40 mg (RhB)/m^2^ for pH1.5-TiO_2_/MoS_2_, pH3.5-TiO_2_/MoS_2_, pH5.5-TiO_2_/MoS_2_, pH7.5-TiO_2_/MoS_2_, pH9.5-TiO_2_/MoS_2_, pH11.5-TiO_2_/MoS_2_, MoS_2_ and CM-TiO_2_, respectively. The result reveals that the increasing order of surface photocatalytic activity is pH1.5-TiO_2_/MoS_2_ < CM-TiO_2_ < MoS_2_ < pH3.5-TiO_2_/MoS_2_ < pH5.5-TiO_2_/MoS_2_ < pH7.5-TiO_2_/MoS_2_ < pH9.5-TiO_2_/MoS_2_ < pH11.5-TiO_2_/MoS_2_, among which pH11.5-TiO_2_/MoS_2_ has the highest surface photocatalytic activity. Based on the previous TEM analysis, the co-exposed crystal facets of anatase TiO_2_ spindle nanocrystals in pH5.5-TiO_2_/MoS_2_, pH7.5-TiO_2_/MoS_2_, pH9.5-TiO_2_/MoS_2_, and pH11.5-TiO_2_/MoS_2_ composites are mainly {101}, {001} and {010} facets, while the co-exposed facets of the anatase TiO_2_ cuboid crystals in the pH3.5-TiO_2_/MoS_2_ composite are mainly {101} and [111]-facets. It is reported that the {010} facets of anatase TiO_2_ have favorable surface atomic structure and surface electronic structure compared with the {101}, {001} and [111]-facets, and the cooperative mechanism existing on {010} facets is beneficial to the improvement of its reactivity [38]. In view of this, the surface photocatalytic activity of TiO_2_ spindle nanocrystals with co-exposed {101}, {001} and {010} facets (pH5.5-TiO_2_/MoS_2_, pH7.5-TiO_2_/MoS_2_, pH9.5-TiO_2_/MoS_2_, and pH11.5-TiO_2_/MoS_2_) is higher than that of anatase TiO_2_ cuboid crystals with co-exposed {101} and [111]-facets (pH3.5-TiO_2_/MoS_2_).

**Figure 11:**
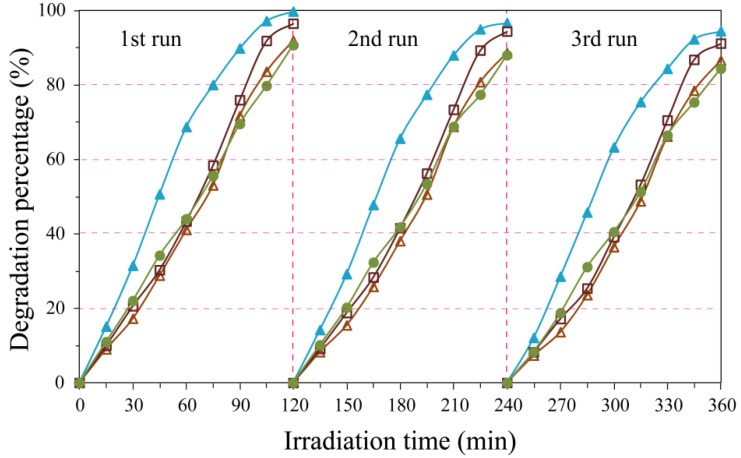
Stability and recyclability test of ultraviolet light irradiation-induced photocatalytic degradation of rhodamine B solution over the as-prepared pH3.5-TiO_2_/MoS_2_, pH5.5-TiO_2_/MoS_2_, pH7.5-TiO_2_/MoS_2_, and pH9.5-TiO_2_/MoS_2_ composites.

Recycling and stability test was measured by carrying out recycling reactions three times for the photocatalytic degradation of RhB over pH3.5-TiO_2_/MoS_2_, pH5.5-TiO_2_/MoS_2_, pH7.5-TiO_2_/MoS_2_, and pH9.5-TiO_2_/MoS_2_ composites, as shown in Figure 11. After recycling three times, the four TiO_2_/MoS_2_ composites still maintain good stability and durability, and the degradation order of RhB is still pH3.5-TiO_2_/MoS_2_ (94.43%) > pH7.5-TiO_2_/MoS_2_ (91.13%) > pH5.5-TiO_2_/MoS_2_ (86.52%) > pH9.5-TiO_2_/MoS_2_ (84.38%).

## 4. Conclusions

In summary, TiO_2_/MoS_2_ composites composed of anatase TiO_2_ nanorods with co-exposed {101} and [111]-facets (pH1.5-TiO_2_/MoS_2_), anatase TiO_2_ nanocuboids with co-exposed {101} and [111]-facets (pH3.5-TiO_2_/MoS_2_), anatase TiO_2_ nanospindles with co-exposed {010} and {101} facets and MoS_2_ microsphere constructed by layer-by-layer self-assembly of nanosheets were synthesized via a facile hydrothermal synthesis method under different pH conditions. The structure, morphology, microstructure, chemical composition, and optical properties of the pH*x*-TiO_2_/MoS_2_ composites were investigated. Additionally, the photocatalytic activity for the degradation of RhB under ultraviolet light irradiation was also investigated, and compared with that of CM-TiO_2_ and MoS_2_. The pH3.5-TiO_2_/MoS_2_ composite exhibited the highest photocatalytic degradation rate, which may be attributed to the synergistic effects of its large specific surface area, suitable heterojunction structure, and favorable photogenerated charge-separation efficiency. Hopefully, this work can provide significant insights into the photocatalytic effect of TiO_2_/MoS_2_ composite with co-exposed high-energy facets and make a contribution to designing more efficient and stable photocatalysts.

## Figures and Tables

**Table 1:** The degradation rate values of RhB with irradiation time.

Samples	Degradation Percentage (%)								
	0 min	15 min	30 min	45 min	60 min	75 min	90 min	105 min	120 min
pH1.5-TiO_2_/MoS_2_	0	9.55	19.95	32.47	44.40	55.33	64.77	72.68	82.33
pH3.5-TiO_2_/MoS_2_	0	15.11	31.46	50.64	68.71	79.99	89.79	97.17	99.70
pH5.5-TiO_2_/MoS_2_	0	9.01	17.25	28.80	41.07	53.03	71.65	83.56	91.86
pH7.5-TiO_2_/MoS_2_	0	9.96	20.59	30.29	43.33	58.37	75.92	91.84	96.46
pH9.5-TiO_2_/MoS_2_	0	10.99	22.02	34.19	44.00	55.57	69.52	79.75	90.68
pH11.5-TiO_2_/MoS_2_	0	10.23	22.91	31.78	42.71	50.28	62.71	76.59	87.67
MoS_2_	0	6.03	11.86	15.32	19.44	21.82	25.17	29.35	33.47
CM-TiO_2_	0	10.77	23.39	31.86	41.44	46.50	52.85	59.44	67.31
Blank	0	2.23	3.37	4.62	7.09	7.57	7.61	8.02	8.87

## Data Availability

The processed data required to reproduce these findings are available by e-mail to the corresponding author.

## References

[1] Chao Y.G., Zheng J.F., Chen J.Z., Wang Z.J., Jia S.P., Zhang H.X., Zhu Z.P. (2017). High efficient visible light-driven hydrogen production of the precious metal-free hybrid photocatalyst: CdS@NiMoS core-shell nanorods. Catal. Sci. Technol..

[2] Matos J., Miralles-Cuevas S., Ruiz-Delgado A., Oller I., Malato S. (2017). Development of TiO_2_-C photocatalysts for solar treatment of polluted water. Carbon.

[3] Chen W.W., Yu S., Zhong Y.Q., Fan X.B., Wu L.Z., Zhou Y. (2018). Effect of electron transfer on the photocatalytic hydrogen evolution efficiency of faceted TiO_2_/CdSe QDs under visible light. New J. Chem..

[4] Verma R., Gangwar J., Srivastava A.K. (2017). Multiphase TiO_2_ nanostructures: A review of efficient synthesis, growth mechanism, probing capabilities, and applications in bio-safety and health. RSC Adv..

[5] Yin Z.Y., Wang Z., Du Y.P., Qi X.Y., Huang Y.Z., Xue C., Zhang H. (2012). Full solution-processed synthesis of all metal oxide-based tree-like heterostructures on fluorine-doped tin oxide for water splitting. Adv. Mater..

[6] Lin Y., Ren P.Y., Wei C.Y. (2019). Fabrication of MoS_2_/TiO_2_ heterostructure with enhanced photocatalytic activity. Cryst. Eng. Comm..

[7] Wei T.C., Lau W.M., An X.Q., Yu X.L. (2019). Interfacial charge transfer in MoS_2_/TiO_2_ heterostructured photocatalysts: The impact of crystal facets and defects. Molecules.

[8] Sabarinathan M., Harish S., Archana J., Navaneethan M., Ikedab H., Hayakawa Y. (2017). Highly efficient visible-light photocatalytic activity of MoS_2_-TiO_2_ mixtures hybrid photocatalyst and functional properties. RSC Adv..

[9] Cai Y., Feng Y.P. (2016). Review on charge transfer and chemical activity of TiO_2_: Mechanism and applications. Prog. Surf. Sci..

[10] Shen M., Yan Z.P., Yang L., Du P.W., Zhang J.Y., Xiang B. (2014). MoS_2_ nanosheet/TiO_2_ nanowire hybrid nanostructures for enhanced visible-light photocatalytic activities. Chem. Commun..

[11] Parzinger E., Miller B., Blaschke B., Garrido J.A., Ager J.W., Holleitner A., Wurstbauer U. (2015). Photocatalytic stability of single- and few-layer MoS_2_. ACS Nano.

[12] Chen B., Meng Y.H., Sha J.W., Zhong C., Hua W.B., Zhao N.Q. (2018). Preparation of MoS_2_/TiO_2_ based nanocomposites for photocatalysis and rechargeable batteries: Progress, challenges, and perspective. Nanoscale.

[13] Bai S., Wang L.M., Chen X.Y., Du J.T., Xiong Y.J.Y. (2015). Chemically exfoliated metallic MoS_2_ nanosheets: A promising supporting co-catalyst for enhancing the photocatalytic performance of TiO_2_ nanocrystals. Nano Res..

[14] Wang D., Xu Y., Sun F., Zhang Q.H., Wang P., Wang X.Y. (2016). Enhanced photocatalytic activity of TiO_2_ under sunlight by MoS_2_ nanodots modification. Appl. Surf. Sci..

[15] Zhang X., Shao C.L., Li X.H., Miao F.J., Wang K.X., Lu N., Liu Y.C. (2016). 3D MoS_2_ nanosheet/TiO_2_ nanofiber heterostructures with enhanced photocatalytic activity under UV irradiation. J. Alloys Compd..

[16] Zhang J., Huang L.H., Lu Z.D., Jin Z.L., Wang X.Y., Xu G.L., Zhang E.P., Wang H.B., Kong Z., Xi J.H. (2016). Crystal face regulating MoS_2_/TiO_2_ (001) heterostructure for high photocatalytic activity. J. Alloys Compd..

[17] Li W.W., Zhao Y., Yuan S.H., Shi L.Y., Wang Z.Y., Fang J.H., Zhang M.H. (2012). Synthesis and characterization of highly dispersed TiO_2_ nanocrystal colloids by microwave-assisted hydrothermal method. J. Mater. Sci..

[18] Wen P.H., Itoh H., Tang W.P., Feng Q. (2007). Single nanocrystals of anatase-type TiO_2_ prepared from layered titanate nanosheets: Formation mechanism and characterization of surface properties. Langmuir.

[19] Wei X.X., Cui B.Y., Wang X.X., Cao Y.Z., Gao L.B., Guo L.B., Chen C.M. (2019). Tuning the physicochemical property of BiOBr via solvent adjustment: Towards an efficient photocatalytic for water treatment. CrystalEngComm.

[20] Peng Y.P., Lo S.L., Ou H.H., Lai S.W. (2010). Microwave-assisted hydrothermal synthesis of *N*-doped titanate nanotubes for visible-light-responsive photocatalysis. J. Hazard. Mater..

[21] Yang W.G., Xu Y.Y., Tang Y., Wang C., Hu Y.J., Huang L., Liu J., Luo J., Guo H.B., Chen Y.G. (2014). Three-dimensional self-branching anatase TiO_2_ nanorods: Morphology control, growth mechanism and dye-sensitized sollar cell application. J. Mater. Chem. A.

[22] Yu J.G., Wang G.H., Cheng B., Zhou M.H. (2007). Effect of hydrothermal temperature and time on the photocatalytic activity and microstructure of bimodal mesoporous TiO_2_ powders. Appl. Catal. B Environ..

[23] Fu W.W., Li G.D., Wang Y., Zeng S.J., Yan Z.J., Wang J.W., Xin S.G., Zhang L., Wu S.W., Zhang Z.T. (2018). Facile formation of mesoporous structured mix-phase (anatase/rutile) TiO_2_ with enhanced visible light photocatalytic activity. Chem. Commun..

[24] Hu Y.D., Chen G., Li C.M., Zhou Y.S., Sun J.X., Hao S., Han Z.H. (2016). Fabrication of {010} facet dominant BiTaO_4_ single crystal nanoplates for efficient photocatalytic performance. J. Mater. Chem. A.

[25] Lu C.X., Liu W.W.H., Li H., Tay B.K. (2014). A binder-free CNT network-MoS_2_ coposite as a high performance anode material in lithium ion batteries. Chem. Commun..

[26] Yousaf A.B., Imran M., Farooq M., Kasak P.P. (2018). Synergistic effect of Co-Ni co-bridging with MoS_2_ nanosheets for enhanced electrocatalytic hydrogen evolution reactions. RSC Adv..

[27] Liu X.G., Du G.R., Li M. (2019). Ture photoreactivity origin of Ti^3+^-doped anatase TiO_2_ crystals with respectively dominated exposed {001}, {101}, and {100} facets. ACS Omega.

[28] Li T., Shen Z.L., Shu Y.L., Li X.G., Jiang C.J., Chen W. (2019). Facet-dependent evolution of surface defects in anatase TiO_2_ by thermal treatment: Implications for environmental applications of photocatalysis. Environ. Sci. Nano.

[29] Song G.S., Luo C.Z., Fu Q., Pan C.X. (2016). Hydrothermal synthesis of the novel rutile-mixed anatase TiO_2_ nanosheets with dominant {001} facets for high photocatalytic activity. RSC Adv..

[30] Han H., Kim K.M., Lee C.W., Lee C.S., Pawar R.C., Jones J.L., Hong Y.R., Ryu J.H., Song T., Kang S.H. (2017). Few-layered metallic 1T-MoS_2_/TiO_2_ with exposed (001) facets: Two-dimensional nanocomposites for enhanced photocatalytic activities. Phys. Chem. Chem. Phys..

[31] Saeed M., Khan I., Adeel M., Akram N., Muneer M. (2022). Synthesis of a CoO-ZnO photocatalyst for enhanced visible-light assisted photodegradation of methylene blue. New J. Chem..

[32] Yasin M., Saeed M., Muneer M., Usman M., Haq A.U., Sadia M., Altaf M. (2022). Development of Bi_2_O_3_-ZnO heterostructure for enhanced photodegradation of rhodamine B and reactive yellow dyes. Surf. Interfaces.

[33] Wang C.X., Lin H.H., Wu J.P., Xu Z.Z., Zhang C. (2016). Controlled formation of TiO_2_ /MoS_2_ core—Shell heterostructures with enhanced visible-light photocatalytic activities. Part. Part. Syst. Charact..

[34] Wu T.X., Liu G.M., Zhao J.C. (1998). Photoassited degradation of dye pollutants. v. self-photosensitized oxidative transformation of *Rhodamine B* under visible light irradiation in aqueous TiO_2_ dispersions. J. Phys. Chem. B.

[35] Wang P., Zhai Y.M., Wang D.J., Dong S.J. (2011). Synthesis of reduced grapheme oxide-anatase TiO_2_ nanocomposite and its improved photoinduced charge properties. Nanoscale.

[36] Wang D.H., Jia L., Wu X.L., Lu L.Q., Xu A.W. (2012). One-step hydrothermal synthesis of *N*-doped TiO_2_/C nanocomposites with high visible light photocatalytic activity. Nanoscale.

[37] Suprabha T., Roy H.G., Thomas J., Kumar K.P., Mathew S. (2009). Microwave-assisted synthesis of titania nanocubes, nanospheres and nanorodes for photocatalytic dye degradation. Nanoscale Res. Lett..

[38] Pan J., Liu G., Lu G.Q., Cheng H.M. (2011). On the true photoreactivity order of {001}, {010}, and {101} facets of anatase TiO_2_ crystals. Angew. Chem. Int. Ed..

